# Hypokalemia as a responsible factor related with the severity of hepatic encephalopathy: a wide multination cross-sectional study

**DOI:** 10.1097/MS9.0000000000000470

**Published:** 2023-06-23

**Authors:** Himayat Ullah, Hossam Shabana, Mohamed Akl Rady, Eman Abdelsameea, Mohamed I. Youssef, Housam Ahmed Helmy, Ghulam Mustafa, Tamer Samir Abd Elghafar, Feras Almarshad, Abdulrahman Alshahrani, Mohamed Abdel-Samiee, Abdulmajeed Ahmed Alwadai, Hendawy Abdel-moety Zedan, Farag khalil, Mahmoud Osama Ahmed, Arafat Kassem, Marwa M. Omar, Shimaa Y Kamel, Saad El Deen Mohamed El sheref, Mohamed Hassan Attia Hassan, Hind S. AboShabaan, Wagih Elgendy, Amr M. Zaghloul, Ibrahim Ghoniem Ramadan Mohamed, Esam Zayed, Amir Abdelghaffar, Galal Abdelhameed Aboufarrag, A. S. Seif, Hussein Ahmed Elmahdy, Ashraf Said, Ali Farahat, Hesham El-Sayed Lashin, Essam Elmahdi, Ahmed Abuamer

**Affiliations:** a Department of Internal Medicine; b Medical Student, College of Medicine, Shaqra University, Dawadmi, Saudi Arabia; Departments of c Internal Medicine; d Department of Hepatology and Gastroenterology, National Liver Institute, Menoufia University, Shebin El-Kom; e Departments of Hepatology, gastroenterology and cinfectious diseases, Al-Azhar University; f Department of Tropical Medicine, Faculty of Medicine, Ain Shams University, Cairo; g Department of Internal Medicine, Faculty of Medicine, Al-Azhar University, Damietta; h Neurology, Faculty of Medicine, Al-Azhar University, Cairo; i Department of Clinical Pathology, Faculty of Medicine; j Department of Clinical Pathology, National Liver Institute, Menoufia University; k Department of Tropical Medicine Hepatology and Gastroenterology, ShebinElkom Teaching Hospital, Menoufia; l Department of Gastroenterology, Hepatology and Infectious Diseases, Danshal Teaching Hospital, El-beheira; m Departments of Biochemistry, Faculty of Science, Cairo University, Giza; n Department of Tropical Medicine and Gastroenterology, Sohag University, Sohag; o Department of Internal Medicine, Faculty of Medicine, Mansoura University, Mansoura, Egypt; p Department of Gastroenterology, MTI-Hayatabad Medical Complex, Peshawar, Pakistan

**Keywords:** hepatic encephalopathy, hypokalemia, liver disease, serum potassium levels

## Abstract

Several precipitating factors of hepatic encephalopathy have been recognized and studied. Hepatic encephalopathy which is a frequent and grave complication of liver failure, is associated with multiple biochemical changes like high serum ammonia, mercaptan and phenol levels, low albumin levels and derangements in electrolytes. It is characterized by a range of neuronal and psychological aberrations mainly due to the inability of liver to metabolize different neurotoxic chemicals produced in the body. Hypokalemia is one of the most important findings in hepatic encephalopathy and postulated as a precipitating factor of the condition. The authors aimed to know the frequency of hypokalemia and its relation to the severity of hepatic encephalopathy.

**Methods:**

After taking approval from the hospital ethical review committee, a total of 5000 patients with hepatic encephalopathy were recruited by consecutive sampling. They were interviewed, examined and investigated for serum potassium levels and other precipitating factors of hepatic encephalopathy.

**Results:**

Total of 5000 patients including 3070 (61.4%) males and 1930 (38.6%) females, aging 13 years and above were studied. The frequency of hypokalemia was 78% (3900 patients). Relating the serum potassium level with the severity of hepatic encephalopathy, 1200 (60%) out of 2000 patients with serum potassium below 2.5 mEq/l were in grade 4 (40%) and 800 out of 2000 were in grade 3 encephalopathy. On the other hand, only 700 patients (6.4%) out 1100 with serum potassium above 3.4 mEq/l were in grade 4 encephalopathy.

**Conclusion:**

Hypokalemia is a frequent finding in patients with hepatic encephalopathy and found to be directly related to its severity.

## Introduction

HIGHLIGHTS

- Hepatic encephalopathy is a frequent and grave complication of liver failure or chronic liver disease.
- Several precipitating factors of Hepatic encephalopathy have been recognized and studied. One important factor among these is hypokalemia.
- The frequency of hypokalemia was 78% (3900 patients). Relating the serum potassium level with the severity of Hepatic encephalopathy, 120 (60%) out of 200 patients with serum potassium below 2.5 mEq/l were in grade 4 (40%) and 80 out of 200 were in grade 3 encephalopathy.
- Hypokalemia is one of the most prevalent precipitating factor of Hepatic encephalopathy and is directly related to its severity.

Hepatic encephalopathy is a syndrome of disordered mentation secondary to hepatic shunting^1^. The main reason for this shunting is advanced liver disease with portal hypertension leading to the development of collateral circulation^2^. Before labelling a patient with hepatic encephalopathy, other causes of central nervous system, dysfunction should be excluded^3–5^. It is episodic and reversible in most of the cases by proper management^6^. The main clinical features of hepatic encephalopathy based on the severity ranges from subtle memory changes to more severe features like coma. Based on the severity of clinical features hepatic encephalopathy is graded as West Haven Grading System from grade 0 to grade 4^3,7,8^. Grade 0 and grade 1 are named as Covert Hepatic Encephalopathy (CHE) by some researchers, while Grades 2 to 4 as Overt Hepatic Encephalopathy (OHE)^9,10^.

## West Haven grades

Grade 0: Subtle memory, intellect and personality changes. It is subclinical.

Grade 1: Mood changes, sleep changes like inversion of sleep pattern, sluggishness in performing simple mathematical tasks.

Grade 2: Behavioural changes like apathy, disorientation mostly in time, drowsiness, gross decline in performing mental tasks, hepatic flap (Asterixis).

Grade 3: Somnolence, inability to perform any mental task, disorientation in time place and person, confused speech.

Grade 4: Coma

Going through the literature, several precipitating factors for hepatic encephalopathy have been identified, including clinical and biochemical factors. Infection, constipation, electrolyte abnormalities, gastrointestinal bleeding, medications like sedatives, transjugular intrahepatic portosystemic shunting, renal failure are some of these factors^8,11,12^.

Hypokalemia is one of the most common findings in patients with hepatic encephalopathy^8,11^. There are multiple mechanisms that promote hypokalemia in patients with liver cirrhosis both due to the disease itself and its treatment^13^. Most widely and commonly understood and explained mechanisms are higher circulatory aldosterone levels which are common in liver cirrhosis and therapeutically used diuretics for oedema and ascites in these patients, apart from other mechanisms^14^. It is an established fact that hypokalemia is one of the common findings in patients with liver cirrhosis and hepatic encephalopathy but does it affect the severity of hepatic encephalopathy? –is a point of debate. It has been postulated that hypokalemia causes increased production of renal tubular ammonia through an unclear mechanism^15^. Although majority of this ammonia is excreted in urine, some of it is reabsorbed into the circulation raising serum ammonia levels thus precipitating hepatic encephalopathy^16^.

## Objective

The main objective of this study was to evaluate serum potassium levels in patients with hepatic encephalopathy. The other objective was to know the prevalence of hypokalemia in these patients and to establish a relationship of hypokalemia with the severity of hepatic encephalopathy.

## Methods

The cross-sectional study was done in highly specialized international treatment centres well concerned with liver diseases management liver disease management (including Hayatabad Medical Complex, Peshawar, Pakistan; Al-Hussein University hospital, Cairo, Egypt; National Liver Institute hospital, Menoufia, Egypt, Sohag University hospital, Ainshams university hospital, Egyptian teaching hospitals). Five thousand cirrhotic patients of different etiologies, with hepatic encephalopathy were included in this study. The inclusion criteria were patients with chronic liver disease of any aetiology, with hepatic encephalopathy, aging above 12 years old. All patients who were having or suspected to have encephalopathy due to any other reason were excluded from the study. Sample size was calculated by a standardized sample calculator keeping the confidence interval above 95% and margin of error below 5%^17^. Sampling technique was non probability convenience sampling. Hepatic encephalopathy was defined on the basis of clinical features, laboratory and radiologic findings of liver cirrhosis and hepatic encephalopathy, excluding other causes of abnormal mentation, like organic brain diseases due to any other cause, patients on diuretics. Detailed history and examination were done in all the participants. In suspected patients, brain imaging was also performed to exclude any organic or vascular brain injury/disease. Serum potassium level was done from well-equipped hospital laboratory by well-experienced professionals. As per international normal values hypokalemia was defined as serum potassium level below 3.5 mEq/l^18^.

Baseline comorbidities such as the presence of diabetes mellitus, hypertension, precipitating factors for encephalopathy and history of smoking were recorded. The work has been reported in line with the Strengthening the Reporting of Cohort Studies in Surgery (STROCSS) criteria^19^.

An informed written consent was taken from the patients or their attorney depending upon the patient’s mental status. The study was conducted in accordance with Good Clinical Practice guidelines and declaration of Helsinki after approval for publication by the hospital’s Research and Ethical Review Board with reference number 597/HEC/B&PSC/2021, dated 2 February, 2022 and Institutional Review Board of Review Board of National Liver Institute, Menoufia University, Egypt (IRB number 00384/2022).

### Statistical analysis

The data collected were analyzed by MS Excel (Microsoft Corporation) and Statistical Package for Social Sciences version 22 (IBM Corporation). Means, medians, and SDs were calculated for continuous variables like age, serum potassium levels. Univariate and multivariate analysis was done to show the significance of correlation of hypokalemia with severity of hepatic encephalopathy after adjusting the other responsible factors of hepatic encephalopathy like hyponatremia, infection, dehydration, constipation, sedative use and gastrointestinal bleed. Spearman correlation coefficient was also calculated to show the relation of potassium level with severity of hepatic encephalopathy. The data obtained were presented in tabulated and graphical form.

## Results

Five thousand patients, aging 13 years and above, with hepatic encephalopathy from both sexes were included in this study, including 3070 (61.4%) males and 1930 (38.6%) females. The mean (±SD) age was 57.36±17.65. Among these hepatic encephalopathy patients, 1100 (22%) patients were normokalemic and 3900 (78%) patients were hypokalemic.

Table 1 showed age and sex distribution, and also the distribution of normokalemia and hypokalemia among these patients. Table 2 showed the distribution of these patients according to the severity of hepatic encephalopathy according to West Haven grading. The mode of grade of hepatic encephalopathy was West Haven grade 3 in these patients.

**Table 1:** Frequency of hypokalemia and normokalemia with age and sex distribution in patients with hepatic encephalopathy (*n*=5000)

	Hypokalemic				Normokalemic				Total			
	Male		Female		Male		Female		Male		Female	
Age (years)	No.	%Age	No	%Age	No	%Age	No.	%Age	No.	%Age	No.	%Age
13–40	280	5.6	160	3.2	110	2.2	70	1.4	390	7.8	230	4.6
41–60	880	17.6	740	14.8	410	8.2	230	4.6	1290	25.8	970	19.4
>60	1240	24.8	600	12.0	150	3.0	130	2.6	1390	27.8	730	14.6
Total	2400	48.0	1500	30.0	670	13.4	430	8.6	3070	61.4	1930	38.6

Mean age was 57.36±17.65.

**Table 2:** Distribution of patients on the basis of severity of hepatic encephalopathy (West Haven criteria) (*n* = 5000)

Hepatic encephalopathy grade	No. patients	%
Grade 1	480	9.6
Grade 2	1870	37.4
Grade 3	2230	44.6
Grade 4	420	8.4
Total	5000	100

Mode of grade of hepatic encephalopathy is 3.

**Figure 1:**
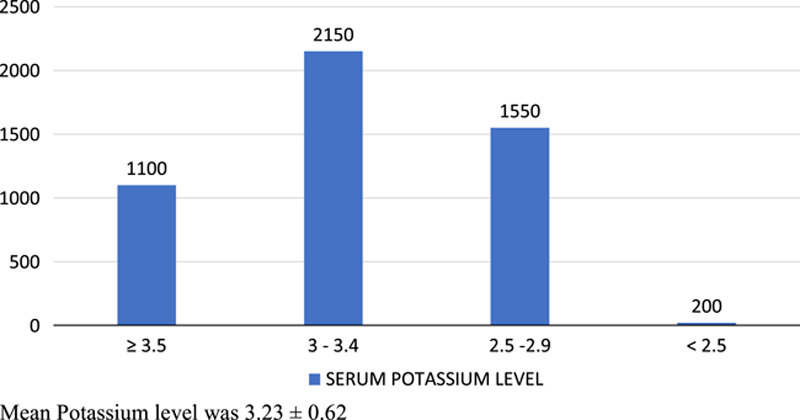
Patients’ distribution according to serum potassium levels (*n*=5000).

**Figure 2:**
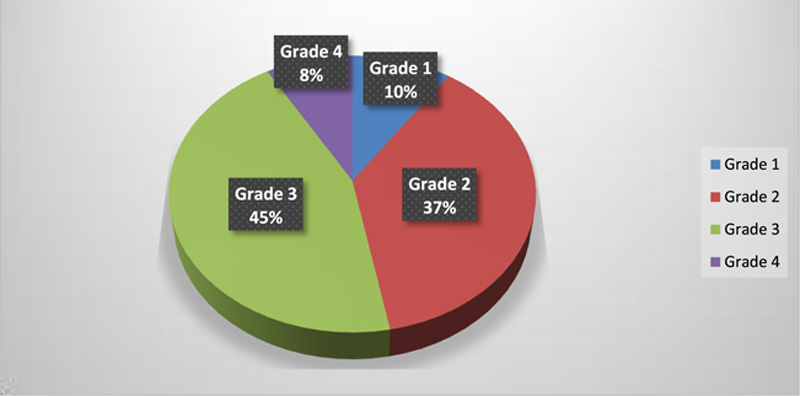
Hepatic encephalopathy grade distribution of patients.

Figure 1 shows distribution of these patients according to the serum potassium level with mean±SD potassium level of 3.23±0.62). Most of these hypokalemic patients were having potassium level ranging between 2.5 and 3.4 mEq/l, which is mild to moderate hypokalemia^18^. Most of the patients with severe hypokalemia (K^+^ <2.5 mEq/l), that is 1200 out of 2000 were in grade 4 hepatic encephalopathy which comprises 60% of patients with severe hypokalemia, while remaining 800 patients were in grade 3 hepatic encephalopathy. Among patients with serum potassium between 2.5 and 2.9 mEq/l: 66.5% of the patients were in grade 3 and 4 encephalopathy while 48% patients with serum potassium of 3–3.4 mEq/l and 34.5% of patients with serum potassium of 3.5 mEq/l and above were in grade 3 and 4 encephalopathy (Fig. 2).

It was found that grade of hepatic encephalopathy was increasing with the fall in the level of serum potassium, with spearman correlation value of −0.01 with serum potassium level, shown in Table 3. Univariate and multivariate analysis corrected for other precipitating factors like hyponatremia, infection, dehydration, constipation, sedative use, gastrointestinal bleeding, also showed statistically significant correlation between level of hypokalemia and hepatic encephalopathy in these patients (*P*<0.01), and shown in Table 4.

**Table 3:** Patients’ distribution according to serum potassium level and grade of hepatic encephalopathy (*n* = 5000)

	Hepatic encephalopathy grade^a^					
Serum potassium level (mEq/l)	Grade 1	Grade 2	Grade 3	Grade 4	Total (% age)	*P* ^*^
≥3.5	200	520	310	70	1100 (22)	0.0001
3–3.4	220	890	980	60	2150 (43)	
2.5–2.9	60	460	860	170	1550 (31)	
<2.5	0	0	80	120	200 (4)	
Total (% age)	480 (9.6)	1870 (37.4)	2230 (44.6)	420 (8.4)	5000 (100)	

^a^ Test used was Chi-square test.

* Spearman rank correlation coefficient was significant at 0.01 level with level of hypokalemia.

**Table 4:** Multivariate analysis (corrected model)

Source	Dependent variable	Type III sum of squares	*df*	Mean square	*F*	Significance	Partial *η* ^2^
Corrected model	SERUM. K	14.709^a^	3	4.903	13.613	0.000	0.076
	SERUM. Na	3801.943^b^	3	1267.314	24.437	0.000	0.129
	Infection	81.156^c^	3	27.052	308.415	0.000	0.651
	Sedative	0.703^d^	3	0.234	12.774	0.000	0.072
	G.I bleed	2.387^e^	3	0.796	14.789	0.000	0.082
	Dehydration	1.047^f^	3	0.349	1.898	0.129	0.011
	Constipation	2.469^g^	3	0.823	8.364	0.000	0.048

^a–g^ The significant correlation between level of hypokalemia and hepatic encephalopathy.

G.I. indicate Gastro Intestinal.

## DISCUSSION

Hypokalemia is one of the most prevalent features in patients with chronic liver disease and hepatic encephalopathy. Whether hypokalemia is just a finding in patients with liver cirrhosis and hepatic encephalopathy or is a precipitant of the condition is still a point of debate among different researchers, even some researchers had shown hyperkalemia as one of a feature of advanced liver cirrhosis^12,20,21^. Our study was aimed at finding the prevalence of hypokalemia in patients with hepatic encephalopathy and then the relation of severity of hypokalemia with the severity of hepatic encephalopathy. It was found that hypokalemia was a very frequent finding in patients with any grade of hepatic encephalopathy, that is 78% of the patients with hepatic encephalopathy were having some degree of hypokalemia. In one of similar study done in the similar settings in a tertiary care hospital, Anwar *et al*.^22^ found that hypokalemia was present in up to 82.6% of the cirrhotic patients with hepatic encephalopathy. Another study done in American college of Gastroenterology, Coukos *et al*.^23^ reported the frequency of hypokalemia to be 75% in patients admitted to ICU due to hepatic encephalopathy.

One of the other findings in our study was the increase in the severity of hepatic encephalopathy with the fall in serum potassium level. In this study, all the patients with potassium level below 2.5 mEq/l (severe hypokalemia) were in grade 3, or 4 encephalopathy, while 66.5% of the patients with serum potassium between 2.5 and 2.9 mEq/l (moderate hypokalemia) were in grade 3 and 4 encephalopathy. The severity of encephalopathy decreased as the serum potassium increased so that only 48% patients of mild hypokalemia and 34.5% of patients with normokalemia were in grade 3 and 4 encephalopathy. Kaplan *et al.* ^24^ reported similar relation between serum potassium level and severity of hepatic encephalopathy, showing that patients with serum potassium less than 3.4 mEq/l had higher rate of hepatic encephalopathy, hepatocellular carcinoma and mortality as compared with those with serum potassium above 3.4 mEq/l.

Since there are many studies that show the association of hypokalemia with severity of hepatic encephalopathy, there are some studies that go against this finding. Cai *et al*.^25^ found in one of their studies that 41.8% of the patients with hyperkalemia were having hepatic encephalopathy against 30.3% of those having normal serum potassium level. But this study was conducted on patients of acute on chronic renal failure.

The brain inflammatory phenotype was characterized by microglial and astrocytic morphological and metabolic alterations (higher oxygen consumption), accumulation of interferon-ɣ and IL-10 in the brain. Recently, improvements in non-invasive assessment of brain oxygenation, perfusion and metabolism using multispectral optoacoustic tomography, MRI, nuclear magnetic resonance spectroscopy and PET imaging, coupled with techniques to assess changes in inflammatory and/or metabolic pathways may help to provide more granular insights on these mechanisms. Most of those tools can be extended into the clinic, to assess evolving disease such as cirrhosis decompensation and acute-on-chronic liver failure, where exacerbation of inflammation and brain dysfunction is clear but the relationship with cerebral perfusion and oxygenation, as well as specific targets for treatment are yet to be elucidated^26,27^.

Our study has clearly showed a high frequency of hypokalemia in patients with hepatic encephalopathy and its deleterious effect on the severity of hepatic encephalopathy. This study may help the clinicians to take prompt actions to manage hypokalemia in order to avoid this serious complication of liver failure.

One of the major advantages of this study was its larger sample size in order to avoid bias.

Our study has some limitations. Options other than hypokalemia for detecting the severity of hepatic encephalopathy were not studied. The main drawback of this study was, it did not study the effects of the corrective measures of hypokalemia on hepatic encephalopathy.

## Conclusion

Hypokalemia is one of the most prevalent findings in patients with hepatic encephalopathy and seems to be directly related to its severity as shown by this and several other studies. So, prompt measures should be taken to avoid and treat this abnormality in order to improve morbidity and mortality in patients with chronic liver disease and liver failure.

## Ethical approval

All study participants were given the opportunity to give their informed consent. Consent taken from patient and his attorney when patient was unable to give consent. The ethical committee reviewed and accepted the report.

## Consent

Consent taken from patient and his attorney when patient was unable to give consent.

## Sources of funding

NA

## Author contributions

All authors contributed to the conceptualization, design, data curation, resource identification, formal analysis and data interpretation. Validation and technique, as well as revision of new software used in the work. All authors shared writing of this workand reviewed the manuscript.

## Conflicts of interest disclosure

The authors declare that there is no conflict of interest regarding the publication of this paper.

## Guarantor

Mohamed Abdel-Samiee.

## Availability of data and material

All data are available upon request.

## Provenance and peer review

Not commissioned, externally peer-reviewed.

## Acknowledgement

The authors thank the Deanship of Scientific Research Shaqra University for supporting this work.

## References

[1] Schenker S Bay MK . Portal systemic encephalopathy. Clin Liver Dis 1997;1:157–184. 15562675 10.1016/s1089-3261(05)70262-2

[2] Riggio O Efrati C Catalano C . High prevalence of spontaneous portal-systemic shunts in persistent hepatic encephalopathy: a case-control study. Hepatology 2005;42:1158–1165. 16250033 10.1002/hep.20905

[3] Patidar KR Bajaj JS . Covert and overt hepatic encephalopathy: diagnosis and management. Clin Gastroenterol Hepatol 2015;13:2048–2061. 26164219 10.1016/j.cgh.2015.06.039 PMC4618040

[4] Luo M Guo JY Cao WK . Inflammation: a novel target of current therapies for hepatic encephalopathy in liver cirrhosis. World J Gastroenterol 2015;21:11815–11824. 26557005 10.3748/wjg.v21.i41.11815 PMC4631979

[5] Butterworth RF . Neurosteroids in hepatic encephalopathy: novel insights and new therapeutic opportunities. J Steroid Biochem Mol Biol 2016;160:94–97. 26589093 10.1016/j.jsbmb.2015.11.006

[6] Wijdicks EFM . Hepatic encephalopathy. N Engl J Med 2016;375:1660–1670. 27783916 10.1056/NEJMra1600561

[7] Weissenborn K . Hepatic encephalopathy: definition, clinical grading and diagnostic principles. Drugs 2019;79(suppl 1):5–9. 30706420 10.1007/s40265-018-1018-z PMC6416238

[8] Cash WJ McConville P McDermott E . Current concepts in the assessment and treatment of hepatic encephalopathy. QJM 2010;103:9–16. 19903725 10.1093/qjmed/hcp152

[9] Blei AT Córdoba J . Hepatic encephalopathy. Am J Gastroenterol 2001;96:1968–1976. 11467622 10.1111/j.1572-0241.2001.03964.x

[10] Kappus MR Bajaj JS . Covert hepatic encephalopathy: not as minimal as you might think. Clin Gastroenterol Hepatol 2012;10:1208–1219. 22728384 10.1016/j.cgh.2012.05.026

[11] Sundaram V Shaikh OS . Hepatic encephalopathy: pathophysiology and emerging therapies. Med Clin North Am 2009;93:819–836. 19577116 10.1016/j.mcna.2009.03.009

[12] Bagny A Lawson-Ananissoh LM Kaaga YL . Hepatic encephalopathy: precipitating factors, clinical and evolutionary aspects at the university hospital campus of lome(Togo). J Liver Res Disord Ther 2018;4:141–143.

[13] Jiménez JV Carrillo-Pérez DL Rosado-Canto R . Electrolyte and acid-base disturbances in end-stage liver disease: a physiopathological approach. Dig Dis Sci 2017;62:1855–1871. 28501971 10.1007/s10620-017-4597-8

[14] Vitale G Coscia K Zavatta G . Secondary hyperaldosteronism and liver fibrosis in patients with compensated chronic liver disease or portal hypertension. Eur J Intern Med 2022;99:118–120. 35039214 10.1016/j.ejim.2022.01.020

[15] Han KH . Mechanisms of the effects of acidosis and hypokalemia on renal ammonia metabolism. Electrolyte Blood Pressure 2011;9:45–49. 22438855 10.5049/EBP.2011.9.2.45 PMC3302905

[16] DuBose TD Good DW Hamm LL . Ammonium transport in the kidney: new physiological concepts and their clinical implications. J Am Soc Nephrol 1991;1:1193–1203. 1932632 10.1681/ASN.V1111193

[17] Sample Size Calculator [http://www.raosoft.com/samplesize.html]. 2020.

[18] Pagana KD Pagana TJ Pagana TN . Mosby’s Diagnostic and Laboratory Test Reference. Mosby; 2020.

[19] Agha R Abdall-Razak A Crossley E . for the STROCSS Group. The STROCSS 2019 Guideline: Strengthening the Reporting of Cohort Studies in Surgery. Int J Surg 2019;72:156–165. 31704426 10.1016/j.ijsu.2019.11.002

[20] Alsaad AA Stancampiano FF Palmer WC . Serum electrolyte levels and outcomes in patients hospitalized with hepatic encephalopathy. Ann Hepatol 2018;17:836–842. 30145570 10.5604/01.3001.0012.3144

[21] Maiwall R Kumar S Sharma MK . Prevalence and prognostic significance of hyperkalemia in hospitalized patients with cirrhosis. J Gastroenterol Hepatol 2016;31:988–994. 26598065 10.1111/jgh.13243

[22] Anwar Ali Jamali Ghulam Mustafa Jamali Ameer Ali Jamali . Association of hypokalemia with hepatic encephalopathy in patients of cirrhosis of liver at tertiary care hospital. IAJPS 2018;5:1831–1838.

[23] Coukos J Hakimian S Lui J . Electrolyte abnormalities in ICU patients with hepatic encephalopathy treated with lactulose: 923. Am J Gastroenterol 2017;112:S518–S520.

[24] Kaplan M Ateş I Gökcan H . Prognostic utility of hypokalemia in cirrhotic patients. Acta Gastroenterol Belg 2018;81:398–403. 30350528

[25] Cai JJ Wang K Jiang HQ . Characteristics, risk factors, and adverse outcomes of hyperkalemia in acute-on-chronic liver failure patients. BioMed Res Int 2019;2019:6025726. 30937312 10.1155/2019/6025726 PMC6415283

[26] Moreau R Jalan R Gines P . Acute-onchronic liver failure is a distinct syndrome that develops in patients with acute decompensation of cirrhosis. Gastroenterology 2013;144:1426–1437. 23474284 10.1053/j.gastro.2013.02.042

[27] Mikkelsen ACD Thomsen ACD Mookerjee ACD . The role of brain inflammation and abnormal brain oxygen homeostasis in the development of hepatic encephalopathy. Metab Brain Dis 2022;38:1707–1716. 36326976 10.1007/s11011-022-01105-2

